# Tuning Amphiphilicity of Particles for Controllable Pickering Emulsion

**DOI:** 10.3390/ma9110903

**Published:** 2016-11-08

**Authors:** Zhen Wang, Yapei Wang

**Affiliations:** Department of Chemistry, Renmin University of China, Beijing 100872, China; 2012wangzhen@ruc.edu.cn

**Keywords:** Pickering emulsion, particle-stabilized emulsion, particle amphiphilicity, emulsion stability, emulsion phase inversion

## Abstract

Pickering emulsions with the use of particles as emulsifiers have been extensively used in scientific research and industrial production due to their edge in biocompatibility and stability compared with traditional emulsions. The control over Pickering emulsion stability and type plays a significant role in these applications. Among the present methods to build controllable Pickering emulsions, tuning the amphiphilicity of particles is comparatively effective and has attracted enormous attention. In this review, we highlight some recent advances in tuning the amphiphilicity of particles for controlling the stability and type of Pickering emulsions. The amphiphilicity of three types of particles including rigid particles, soft particles, and Janus particles are tailored by means of different mechanisms and discussed here in detail. The stabilization-destabilization interconversion and phase inversion of Pickering emulsions have been successfully achieved by changing the surface properties of these particles. This article provides a comprehensive review of controllable Pickering emulsions, which is expected to stimulate inspiration for designing and preparing novel Pickering emulsions, and ultimately directing the preparation of functional materials.

## 1. Introduction

Emulsions have been extensively used in many areas such as cosmetics, the food industry, and material science [1,2,3,4,5,6,7]. As a multiphasic mixture system, emulsions typically consist of three main components: oil phase, water phase, and emulsifier [8]. Various emulsifiers, including surfactants [9,10,11,12], polymers [13,14,15,16,17], proteins [18,19,20,21,22,23], and particles [24,25,26,27], have been utilized to prepare different kinds of emulsions. Compared with emulsions stabilized by surfactants, polymers, and proteins, particles stabilized emulsions, which are commonly named Pickering emulsions, enjoy characteristic superiority [2]. They make use of micro- or nano-size particles as the interfacial stabilizers, which provide a robust physical barrier against droplet coalescence and retain the long-term stability of emulsions. In addition, Pickering emulsions have low cytotoxicity and good biocompatibility as a result of reducing the use of surfactants [28]. Pickering emulsions as potential candidates are expected to replace traditional emulsions to some extent, and are receiving much attention from industry, bioscience, and materials. 

The control of Pickering emulsions plays a pivotal part in many significant processes, for example, oil extraction and recovery [29,30], emulsion polymerization [31,32,33,34], and heterogeneous catalysis [35,36,37]. In this regard, many endeavors have been made in developing controllable Pickering emulsions. Generally, the control of Pickering emulsions involves control over their stability and type which determine the property and performance of the emulsions [8,38]. There are two ways to realize this: changing the compositions of the emulsion phases or tailoring the amphiphilicity of the interfacial emulsifiers. Based on the Ostwald packing theory, it is well known that changing the water-to-oil ratio of emulsion systems can give rise to interconversion between different types of emulsions which is commonly named emulsion phase inversion [8], for example, from water-in-oil (W/O) emulsion to oil-in-water (O/W) emulsion. Together with the water-to-oil ratio, the change of many other parameters such as the concentration of Pickering particles or the components of the two liquid phase, can also induce emulsion phase inversion [39]. These strategies of changing the compositions of emulsion systems can effectively control the type and morphology of Pickering emulsions. However, complicated operation processes and repeated experimental procedures are required to establish the optimum condition for the formation of the desired Pickering emulsions, which cause vast time expenditure and material waste. On the other hand, the addition of excess components including oil phase, water phase, and Pickering particles may damage the original stability of emulsions and lead the emulsions to undergo irreversible changes.

The amphiphilicity of emulsifiers is another crucial factor that notably affects the emulsion stability and type [40,41,42,43]. In surfactant stabilized emulsion systems, the amphiphilicity of surfactants is defined as the relative balance between hydrophilic and hydrophobic properties of amphiphiles, which can be well described by the hydrophilic-lipophilic balance (HLB) value [2,8,44]. However, the amphiphilicity in Pickering emulsion systems has different definitions depending on the types of particle. For isotropic particles such as rigid and soft spherical particles, the surface wettability is widely used to describe the amphiphilicity of particles. It can be measured by the three-phase contact angle of particles adsorbed at an oil-water interface [2,44,45]. For anisotropic Janus particles, the concept of Janus balance has been proposed to represent the amphiphilicity of particles in some studies [46,47]. The change of surface wettability or Janus balance can lead to the change of amphiphilicity, which is routinely used to manipulate the stability and type of Pickering emulsions. In contrast to changing the compositions of emulsion systems, tuning amphiphilicity of Pickering particles can direct not only phase inversion between different types of Pickering emulsions, but also reversible emulsification and demulsification via an easier regulating process. This pathway for building controllable Pickering emulsions, as well as their functional materials, is becoming a front-burner issue in the field. 

In this review, we highlight some recent advances in tuning the amphiphilicity of particles for preparing controllable Pickering emulsions. Based on the difference of particle species, Pickering emulsifiers are classified into three categories: rigid particles, soft particles, and Janus particles. In the section of rigid particles, we discuss in detail the isotropic rigid particles that are used to control the stability and type of Pickering emulsions. Different mechanisms of tailoring the amphiphilicity of particles are summarized, including molecular adsorption and chemical grafting of small molecules and polymers. For soft particles, examples of isotropic self-assembled objects and microgels are summarized. The next section presents some anisotropic Janus particles that have been successfully used in controlling Pickering emulsions. The amphiphilicity of Janus particles is tuned by changing their surface chemistry and shape. This review is expected to stimulate interest in controlling Pickering emulsion by tuning the amphiphilicity of particles, and to make a contribution in extending the research scope of controllable Pickering emulsions and their future applications in many fields. 

## 2. Rigid Particles

Various unmodified rigid particles have been attempted as emulsifiers to prepare Pickering emulsions in recent years, for example, silica spheres and rods [48,49,50], metal oxide particles [51,52], clay particles [53], graphene nanosheets [54,55,56], carbon nanotubes [57,58], carbon black [59], cellulose nanocrystals [60,61], polymeric particles [62,63], lignin particles [64], and chitin particles [65,66]. Stable Pickering emulsions can be successfully formed by using some of these particles. Additionally, the obtained Pickering emulsions have different types depending on the inherent surface wettability of particles. The more hydrophilic particles favor the formation of oil-in-water (O/W) emulsions and the more hydrophobic particles always conduct water-in-oil (W/O) emulsions [2]. However, the particles with specific surface wettability can only form one type of emulsion by keeping the emulsion compositions unchanged. In the absence of stimuli-responsive groups on the surface of particles, it is almost impossible to actualize phase inversion by only using those unmodified particles. For other particles with extremely hydrophilic or hydrophobic surfaces, they generally tend to be dispersed in water or oil phase instead of adsorbing at the oil-water interface, which always causes the emulsification failure or instability of Pickering emulsions. These existing limitations restrict control over Pickering emulsions. In order to easily control the stability and type of Pickering emulsions, tuning the amphiphilicity referring to the controllable change of surface wettability is required for the rigid particles. Among the present strategies for tuning the particle amphiphilicity [28], surface modification is an effective method and has been extensively made use of. According to the interaction mode between particle surface and particular small molecules or polymers, this is generally classified into two categories, including surface adsorption and surface grafting. In this section, we review recent progress related to the two strategies for tuning the amphiphilicity of rigid particles for controllable Pickering emulsions.

### 2.1. Surface Modification by Non-Covalent Adsorption

#### 2.1.1. Small Molecules

**Figure 1:**
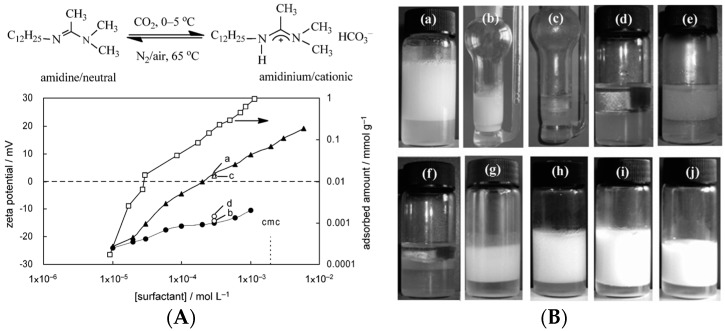
(**A-top**) Schematic illustration of the interconversion between neutral amidine form and cationic amidinium form of N′-dodecyl-N,N-dimethylacetamidine; (**A-bottom**) Zeta potentials of silica nanoparticles that were dispersed in aqueous solutions containing switchable surfactant with cationic amidinium form (▲), and neutral amidine form (●), as a function of initial concentration. The adsorption isotherm of the amidinium form (□) at the silica–water interface is also given as a function of the equilibrium concentration; (**B**) Digital photographs of n-octane-in-water Pickering emulsions that were stabilized by silica nanoparticles and either a switchable surfactant (a–h) undergoing switching or cetyltrimethylammonium bromide (CTAB) (i,j). (**a**) Emulsion with amidinium; (**b**) transfer to a bubbling device; (**c**) bubbling of N_2_ through the emulsion; (**d**) transfer to a vial; (**e**) re-homogenization, 24 h later; (**f**) one week later; (**g**) bubbling CO_2_ through the emulsion, re-homogenization, one week later; (**h**) emulsion with amidinium after 24 h without bubbling of N_2_; (**i**) emulsion with CTAB, 24 h later; (**j**) emulsion with CTAB after bubbling N_2_ through the emulsion, 24 h later. Adapted with permission from [75]. Copyright 2013, WILEY-VCH Verlag GmbH & Co. KGaA, Weinheim, Germany.

**Figure 2:**
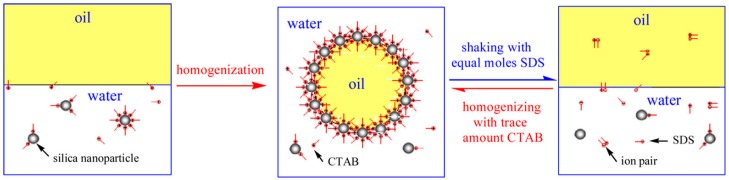
The switch between stable and unstable oil-in-water (O/W) emulsions induced by the alternate addition of a cationic surfactant and an anionic surfactant in the aqueous phase. Reprinted with permission from [76]. Copyright 2015, American Chemical Society.

A variety of ingredients have been developed to modify the surface of rigid particles by molecular adsorption of small molecules and polymers based on non-covalent interactions. Among them, surface modification by small molecule adsorption is simple and has attracted great attention from many researchers [53,67,68]. Numerous examples have been presented in which small molecule adsorption was used to tune the amphiphilicity of particles for controlling the stability of Pickering emulsions. For example, Li and coworkers [69] used the adsorption of short-chain aliphatic amines to change the amphiphilicity of Laponite particles, leading to a stable Pickering emulsion. The unmodified Laponite particles are extremely hydrophilic, unable to stabilize the oil-water interface. Therefore, complete phase separation of paraffin oil and water was observed when the raw Laponite particles were used as emulsifiers to prepare Pickering emulsions. Short-chain aliphatic amines including diethylamine (DEA) and trimethylamine (TEA) can adsorb onto the surface of Laponite particles instead of self-assembling into aggregates in solution. Their absorption can effectively increase the hydrophobicity of Laponite particles, facilitating the enrichment of particles at the O/W interface within the emulsion system. The stability of Pickering emulsions could be regulated by controlling the amine concentration. Similar studies have also been demonstrated, for example, methyl orange-modified Laponite particles [70], palmitic acid-modified silica nanoparticles [71], oleic acid-modified silica nanoparticles [72], and magnetite nanoparticles [73], octyl gallate-modified aluminum oxide particles [74] etc. In addition to steady molecules, stimuli-responsive small molecules can transform their molecular structures or conformations under specific external stimuli [9], which enables the smart control of Pickering emulsions. Jiang et al. demonstrated stabilization and destabilization of O/W emulsions by using N′-dodecyl-N,N-dimethylacetamidine modified silica particles [75]. N′-dodecyl-N,N-dimethylacetamidine is a switchable surfactant with a long-chain alkyl group. At high CO_2_ concentration, it can be protonated to become positively charged, which can adsorb onto the negatively charged silica particles based on electrostatic interaction. As shown in Figure 1, the molecular adsorption was indicated by the zeta potential of the particles and the adsorption isotherm. The modification of the hydrophobic alkyl group resulted in the wettability change of silica particles from excessive hydrophilicity to partial hydrophobicity, allowing the formation of stable O/W emulsions. Upon purging N_2_ or air, the protonated surfactants returned to neutral form and desorbed from the silica particles surface. As a result, the Pickering emulsion was destabilized and phase separation occurred (Figure 1c). Recently, another switchable Pickering emulsion was reported by Zhu and coworkers [76]. As shown in Figure 2, they used a cationic surfactant of cetyltrimethylammonium bromide (CTAB) to change the surface wettability of negatively charged silica particles by electrostatic adsorption. The adsorption of CTAB endowed hydrophilic silica particles with a certain amphiphilicity, which gave a generation of stable O/W emulsions. Subsequently, the obtained Pickering emulsions were destabilized with the addition of an anionic surfactant of sodium dodecyl sulfate (SDS) due to the formation of ion pairs and desorption of CTAB from the particle surface. The stable O/W emulsion could be formed again by adding an equimolar amount of CTAB. The alternate addition of CTAB and SDS in aqueous solution reversibly tuned the amphiphilicity of the silica particles, thus ensuring the control of the destabilization-stabilization behavior of the emulsions.

**Figure 3:**
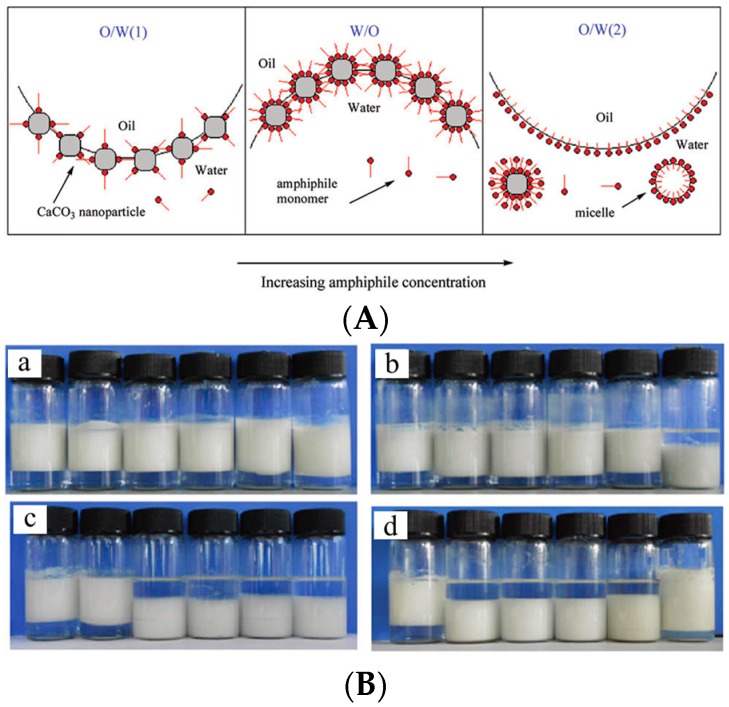
(**A**) Schematic illustration of phase inversion induced by surface adsorption of a series of sodium carboxylates of different chain lengths on CaCO_3_ nanoparticles; (**B**) Digital photographs of vessels containing toluene-water emulsions stabilized by CaCO_3_ nanoparticles and sodium carboxylate: (**a**) C_6_Na; (**b**) C_8_Nal; (**c**) C_10_Na; and (**d**) C_12_Na at different concentrations, taken 1 week after preparation. Concentration from left to right: (a–c) 1, 3, 6, 10, 30, 60 mM; (d) 1, 3, 10, 30, 100, 300 mM. Adapted with permission from [78]. Copyright 2012, American Chemical Society.

The phase inversion of Pickering emulsions could be also realized by surface modification of small molecules. Cui and coworkers [77] demonstrated a successful phase inversion from O/W emulsion to W/O emulsion by using a kind of double-chain cationic surfactant-modified silica particle. It was noted that the unmodified silica particles were unable to stabilize the O/W emulsions due to their excessive hydrophilicity. The single-chain cationic surfactants such as dodecyltrimethylammonium bromide (DTAB) and CTAB can adsorb onto the silica particles’ surface, therefore increasing the surface hydrophobicity. The adsorption of DTAB or CTAB could improve the stability of O/W emulsions, but the change of particle amphiphilicity was not enough to curve the interface of emulsion into W/O form because of insufficient hydrophobicity of the particles’ surface. Compared with single-chain cationic surfactant, a double-chain cationic surfactant of didodecyldimethylammonium bromide (di-C_12_DMAB) increases the adsorption density of hydrophobic alkyl chains on the particle surface and endows the particle with enhanced hydrophobicity. Their adsorption ultimately induced the emulsion phase inversion from O/W emulsion to W/O emulsion. After that, a similar behavior [78] was observed again by the same group while using CaCO_3_ nanoparticles and a series of sodium carboxylates with different chain lengths to stabilize Pickering emulsions (Figure 3). The sodium carboxylates have hydrophilic head groups with negative charges and hydrophobic alkyl tails. They can adsorb onto the positively charged CaCO_3_ nanoparticles’ surface based on the combination of electrostatic interaction and hydrophobic effect. With the increase of alkyl chain length, they show enhanced adsorption ability on the particle surface. The adsorbed sodium carboxylates form a monolayer on the CaCO_3_ nanoparticles with head groups to the particles’ surface and hydrophobic tails to water. The arrangement of amphiphiles resulted in the increase of the hydrophobicity of the particles’ surface and the change of amphiphilicity. When a low concentration of sodium carboxylate was used, the particle surface reached a certain degree of amphiphilicity, which favored the formation of stable O/W emulsion. As the hydrophobicity was increased to a particular value by increasing the concentration or alkyl chain length of the sodium carboxylates, phase inversion from O/W emulsion to W/O emulsion occurred. Furthermore, for C_12_Na with a long alkyl chain, bilayer or hemimicelle was formed at higher concentration of sodium carboxylates due to the strong chain-chain interactions, which turned the particles hydrophilic again and caused desorption of particles from the oil-water interface. A second phase inversion from W/O emulsion to O/W emulsion was achieved (Figure 3d). 

#### 2.1.2. Polymers

**Figure 4:**
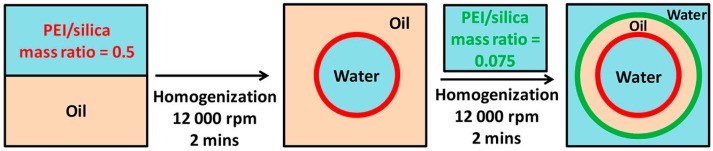
Schematic representation of preparing water-oil-water (W/O/W) double emulsions using poly(ethylene imine)-modified silica particles. Reprinted with permission from [80]. Copyright 2014, American Chemical Society.

As stated in the above section, surfactants or surfactant-like molecules were usually used for tuning the amphiphilicity of particles. However, those molecules may give rise to toxicity issues in consideration of biological applications and have a potential possibility of uncontrolled fast desorption from particle surfaces [2,40,79]. Compared with small molecules, polymers have more advantages in biocompatibility and adsorption stability. Polymers also possess more varied chain conformations and chain behavior, which can facilitate the formation of complex Pickering emulsions such as double Pickering emulsion [80] and high-internal-phase emulsion (HIPE) [81], and fulfil high-level controllability of Pickering emulsions. Various polymers have been applied to change the particle wettability intending to control the Pickering emulsions. For instance, Wang and coworkers [82] improved the stability of paraffin-water emulsions by using poly(oxypropylene)diamine-modified Laponite particles. Either the Laponite particle or poly(oxypropylene)diamine is a poor emulsifier due to the unfavorable amphiphilicity. When one of them was used alone to prepare the paraffin-water emulsion, complete phase separation into oil and water phase took place. The stable O/W emulsion could be only formed by their combination. It was interpreted that poly(oxypropylene)diamine could adsorb on the particle surface with the two end groups anchored on the surface and the hydrophobic poly(oxypropylene) chain exposed to water, thus changing the amphiphilicity of the particles. Williams et al. [80] used silica particles modified with poly(ethylene imine) (PEI) to prepare a double Pickering emulsion. In the absence of PEI, the hydrophilic silica particles were unable to stabilize any type of Pickering emulsion. Upon absorption of PEI on silica particles with a PEI/silica mass ratio of 0.075, the particles turned partially hydrophobic which enabled the formation of stable O/W emulsions. When the PEI/silica mass ratio was further increased to 0.5, the enhanced surface hydrophobicity led to the phase inversion of emulsion from the O/W form to the W/O form. In short, the interface with different curvature could be formed by using the PEI/silica hybrid particles with a different adsorbed amount of PEI. Based on this mechanism, the double Pickering emulsion of the W/O/W form was also prepared by two-step homogenization (Figure 4). A W/O Pickering emulsion was fabricated at high PEI/silica mass ratio through a first homogenization. This obtained W/O emulsion was subsequently applied to generate the final W/O/W double emulsion at low PEI/silica mass ratio through a second homogenization. 

**Figure 5:**
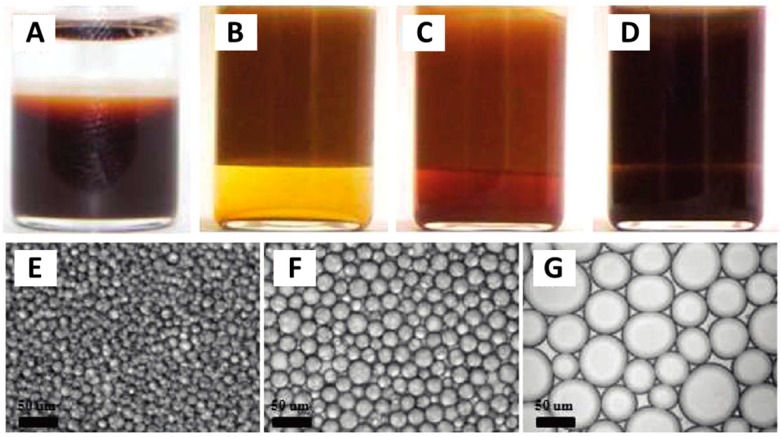
Photographs and microscopy images of oil-in-water emulsions formed between dodecane and aqueous dispersions containing (**A**) poly(acrylic acid) (PAA)-coated NPs; (**B**,**E**) PAA_114_-b-PBA_26_-coated NPs; (**C**,**F**) PAA_114_-b-PBA_38_-coated NPs; and (**D**,**G**) PAA_114_-b-PBA_67_-coated NPs. Images were captured after 1 day at pH = 8 with equal volumes of oil and water phases. Adapted with permission from [84]. Copyright 2012, American Chemical Society.

Block copolymer is another kind of polymer used to tune the amphiphilicity of particles. Binks and coworkers [83] prepared a polymer-particle complex with tunable amphiphilicity by using the poly[2-(dimethylamino)ethyl methacrylate-block-methyl methacrylate] (PDMA-b-PMMA) to adsorb onto latex particles based on hydrophobic effect. The amphiphilicity of the polymer-particle complexes can be tuned by changing the environmental temperature which has a great influence on the degree of hydration of PDMA chains. At low temperature, the polymer-particle complexes were hydrophilic, which can stabilize an O/W emulsion. With the increase of temperature, the degree of hydration of PDMA chains was reduced. The polymer-particle complexes became more hydrophobic and preferentially wetted by oil. The change in surface wettability induced by increasing temperature brought about phase inversions from O/W emulsions to W/O/W emulsions, finally to W/O emulsions which were formed at the higher temperature. Yoon et al. [84] investigated the effect of poly(acrylic acid) (PAA)-based polymers that were adsorbed onto iron oxide nanoparticles on the morphologies of Pickering emulsions. PAA-based polymers can adsorb onto the iron oxide nanoparticles to change their surface amphiphilicty based on coordination interaction between the carboxylate groups of PAA and the iron. Four PAA-based polymers were attempted to tune the amphiphilicity of iron oxide nanoparticles. The adsorption of the homopolymer PAA could not change the hydrophilicity of iron oxide nanoparticles. Only macroscopic phase separation occurred when these particles acted as emulsifiers during the preparation of Pickering emulsions (Figure 5A). The other three PAA-based polymers are block copolymers poly(acrylic acid-b-butyl acrylate) (PAA-b-PBA) with different PBA block lengths. With the decrease of PBA block length, the interfacial tension of particles at the dodecane/water interface was also decreased. This interfacial change indicated that the modified particles exhibited enhanced interfacial activity. Due to the shortest PBA block length, the particles coated with PAA_114_-b-PBA_26_ were expected to have the most appropriate amphiphilicity. As shown in Figure 5E, stable emulsions with small droplet size were successfully generated by using these particles. With the increase of PBA block length, the interfacial activity of the particles was lowered, and more nanoparticles were observed to be dispersed in excess aqueous phase. The change of particles in amphiphilicity and decrease of particles’ concentration cooperatively led to the increase of emulsion droplet size (Figure 5F,G).

### 2.2. Surface Modification by Chemical Grafting

Although molecular adsorption is a simple and effective method to tailor the amphiphilicity of particles, there are still many limitations existing in this method [28]. Molecular adsorption generally has adsorption and desorption equilibrium. This equilibrium crucially relies on the conditions of the system. When the equilibrium conditions are changed, the adsorbed molecules may desorb from the particle surface, which will possibly cause uncontrollable emulsification failure and destabilization of Pickering emulsions. In addition, excessive small molecules or polymers are needed to maintain the equilibrium. The residual molecules, particularly small molecule surfactants or amphiphilic block copolymers, can stabilize the oil-water interface alone. It is difficult to distinguish the exact contributions of the modified particles and the residual molecules to the emulsion stability and phase inversion. The advantages of Pickering emulsion are somehow weakened due to the addition of other amphiphilic molecules. Compared with molecular adsorption, chemical grafting of particular groups on the particle surface is receiving more attention. Chemical grafting requires small molecules or polymers to be fixed on the particle surface by covalent bonds, which ensures less influence against the change of system conditions. Importantly, this strategy can endow particles with various stimuli-responsive groups, for example, temperature, light, pH, CO_2_, and ion strength, which reclaims a new vista to control the emulsion type and stability of Pickering emulsions. Similarly, molecules chemically grafted on the particle surface can be small molecules or polymers. In this next section, some recent studies on tuning the amphiphilicity of particles via chemical grafting are reviewed as below. 

#### 2.2.1. Small Molecules

**Figure 6:**
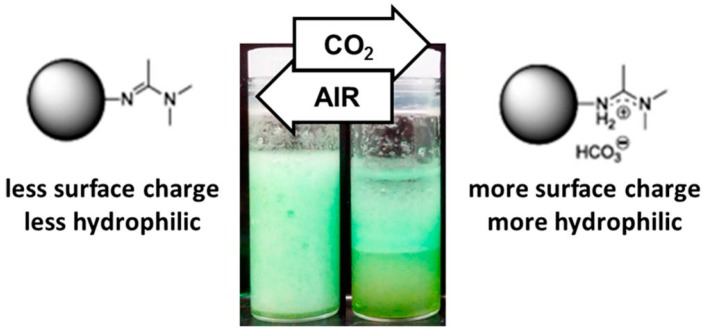
A schematic illustration for demonstrating the change of CO_2_-responsive molecule-modified particle amphiphilicity by bubbling and releasing CO_2_ and the resulting stabilization/destabilization of emulsions. Reprinted with permission from [90]. Copyright 2014, American Chemical Society.

Various small molecules have been grafted onto the particle surface to enable the particles to be stimuli-responsive. The capability of responding to external stimuli allows these modified particles to behave as smart emulsifiers for controlling Pickering emulsions. Depending on the properties of small molecules, different external stimuli can be used to actuate the change of particle amphiphilicity. Among them, the amphiphilicity regulation by pH and ion strength has been extensively reported [85,86,87,88]. Many reviews have highlighted these excellent works [2,28,45]. It is worth mentioning that tuning the amphiphilicity by CO_2_ is becoming a new method. CO_2_ is an attractive stimulus owing to its particular advantages of low cost and good biocompatibility [89]. Importantly, it is completely erasable for the emulsion system without any chemical residue contamination. The stability and phase inversion of the emulsion can be readily controlled by only bubbling and releasing CO_2_. Liang and coworkers [90] manipulated the stability of Pickering emulsion by using CO_2_-responsive particles which were prepared by grafting a CO_2_-responsive small molecule of N,N-dimethylacetamide dimethyl acetal (DMA-DMA) on silica particles. The freshly obtained CO_2_-responsive particles are able to stabilize an O/W emulsion due to their surface amphiphilicity meeting the basic requirement of forming a stable emulsion. Upon bubbling CO_2_ into the emulsion, DMA-DMA was protonated to generate surface charges, and thus the particles became more hydrophilic. This wettability change induced destabilization and phase separation of the emulsion. In contrast, DMA-DMA and phenyl were co-grafted onto silica particles to obtain more hydrophobic particles with completely different initial wettability. Correspondingly, stable W/O emulsions were prepared by these modified particles. When the Pickering emulsion was bubbled with CO_2_, a similar phenomenon of emulsion destabilization was observed. In both systems, the stable O/W or W/O emulsions could be recovered by the removal of CO_2_ via purging with air (Figure 6).

**Figure 7:**
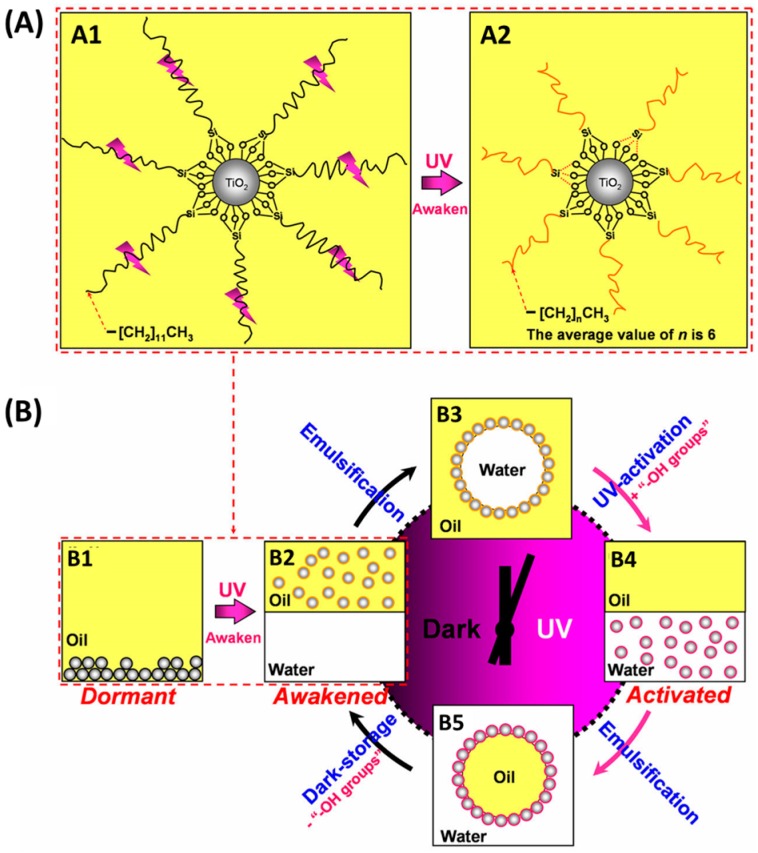
(**A**) Schematic representation of a UV-responsive nanoparticle emulsifier consisting of a TiO_2_ core with a shell of long alkyl chain silanes (**A1**) and degraded to short-chain silanes (**A2**) via UV irradiation in hexane; (**B**) Schematic of the switchable Pickering emulsions using TiO_2_ nanoparticle emulsifiers under UV/dark treatments: (**B1**) the dormant emulsifiers; (**B2**) the emulsifiers awakened via UV irradiation; (**B3**) the stabilized W/O type emulsions; (**B4**) coalescence and phase separation of the W/O emulsions in response to UV-activation; (**B5**) the stabilized O/W type emulsions and (**B2**) coalescence and phase separation of the W/O emulsions induced by dark storage. The oil is hexane. Adapted with permission from [93]. Copyright 2015, American Chemical Society.

Light is another neat and contactless external stimulus [91]. Using light to tune the surface chemistry of particles is considered to be an ideal non-invasive way to control Pickering emulsions, which has been given tremendous attention recently. Chen and coworkers [92] used photochromic spiropyran-grafted up-conversion nanophosphors (Sp-UCNPs) as emulsifiers to establish reversible phase inversion within a Pickering emulsion via light irradiation. In their study, spiropyran was typically grafted on the surface of up-conversion nanophosphors by EDC/NHS chemistry. In terms of conversion of NIR light to UV light by UCNPs, spiropyrans which are responsive to UV light can be triggered from the close-state to the open-state by NIR light. Once photoisomerization occurred, the hydrophobic particles became hydrophilic, leading to the formation of O/W emulsions. Upon the visible light irradiation, spiropyran returned to the close-state so that the particle became hydrophobic again and W/O emulsions were formed. The conversion of emulsion types could be repeated many times by alternating NIR light and visible light irradiation. This novel system was further extended to control biphasic catalysis. Zhang et al. [93] reported a photo-switchable Pickering emulsion system with the use of modified TiO_2_ nanoparticles as emulsifiers. Due to the responsive ability of TiO_2_ to UV light, reversible regulation of particle amphiphilicity could be accomplished by alternative UV/dark treatment. The raw TiO_2_ nanoparticles were grafted with long alkyl chain silanes to change their inherent surface wettability. However, with the amphiphobic property of the particle surface after grafting, the modified TiO_2_ nanoparticles were unable to be wetted by oil or water phase and no emulsion was formed. Upon UV irradiation, the excited TiO_2_ nanoparticles could induce degradation of the alkyl chain into shorter species. This slight change of surface wettability was enough to retain the stability of the W/O emulsions. With further UV irradiation, alkyl groups were further removed and the particles were covered with hydroxyl groups. The dramatic increase of surface hydrophilicity caused phase inversion and yielded O/W emulsions. Keeping in the dark for a period, the particle surface changed to be hydrophobic and the W/O emulsions were formed again. The different types of emulsions were manipulated by using UV light irradiation and dark-storage alternately (Figure 7). 

#### 2.2.2. Polymers

**Figure 8:**
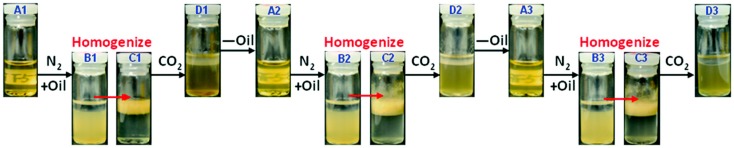
Three cycles of the N_2_/CO_2_-triggered emulsified/demulsified process of lignin-g-PDEAEMA Pickering emulsion. Reprinted with permission from [94]. Copyright 2014, Royal Society of Chemistry.

**Figure 9:**
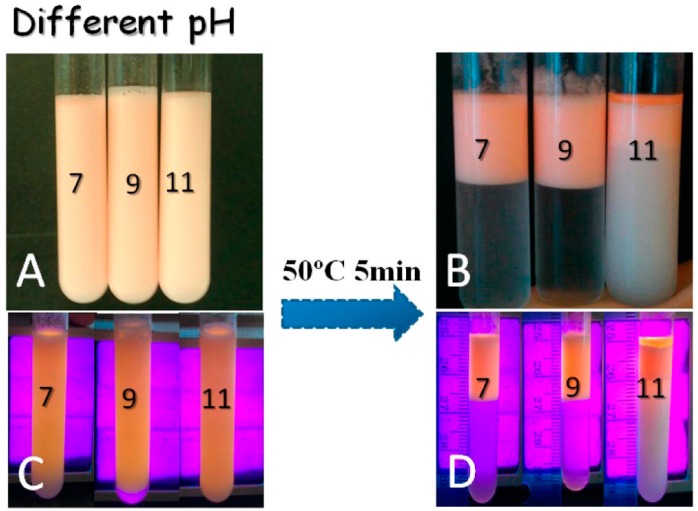
Photographs of toluene-water emulsions stabilized by PDMAEMA-g-CNC at different pH: without UV (**A**); with UV light (**C**); Photographs of emulsions taken after equilibration in a water bath for 5 min (without UV (**B**) and with UV light (**D**)). Reprinted with permission from [95]. Copyright 2014, American Chemical Society.

Polymers can be also grafted on particles to tailor particle amphiphilicity for controllable Pickering emulsions. Qian and coworkers [94] used a CO_2_-responsive poly[2-(diethylamino)ethyl methacrylate] (PDEAEMA) to modify lignin particles through atom transfer radical polymerization (ATRP). The obtained lignin-g-PDEAEMA particles could be dispersed in water in the presence of CO_2_ while being flocculated or even precipitated by purging with N_2_ quickly (Figure 8). For untreated lignin-g-PDEAEMA particles, decane-in-water emulsions could be stabilized for more than one month. With the addition of CO_2_, the O/W emulsion was destabilized into two separated phases. Another two cycles of stabilization and destabilization were also provided by the repeated addition and removal of CO_2_ in this study. In addition to CO_2_, PDEAEMA is also responsive to temperature. This intriguing property also affords great convenience to switch stabilization and destabilization of Pickering emulsions. In this regard, Tang and coworkers [95] grafted PDMAEMA chains onto the surface of cellulose nanocrystals (CNC) via free radical polymerization. The obtained PDMAEMA-g-CNC particles could adsorb at the oil-water interface and remarkably reduce the interfacial tension, which was beneficial for the formation of stable O/W emulsions. Due to the thermo-responsive character, PDMAEMA chains underwent amphiphilicity change from hydrophilicity to hydrophobicity with the increase of temperature. The O/W emulsions formed at a given pH were destroyed and separated into two phases when they were kept at 50 °C for 5 min (Figure 9). A similar behavior was also observed by the same group using poly(oligoethylene glycol) methacrylate (POEGMA) to modify cellulose nanocrystals [96]. Many other stimuli-responsive polymers have also been reported to tailor the amphiphilicity of particles. However, to our knowledge, only a few light-responsive polymers for controllable Pickering emulsions have been investigated up till now. Enormous opportunities still exist for using non-invasive methods to tune the amphiphilicity of particles by polymer grafting. 

## 3. Soft Particles

### 3.1. Self-Assembled Objects

**Figure 10:**
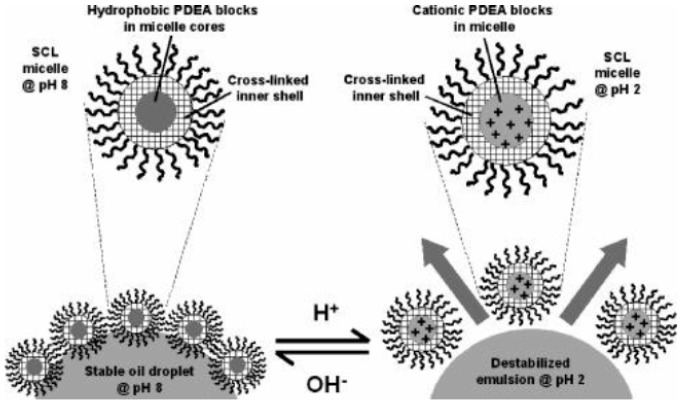
Schematic representation of pH-induced emulsification and demulsification using shell cross-linked micelles as particulate emulsifiers. Dewetting from the oil droplet surface occurs at low pH. Reprinted with permission from [103]. Copyright 2005, American Chemical Society.

**Figure 11:**
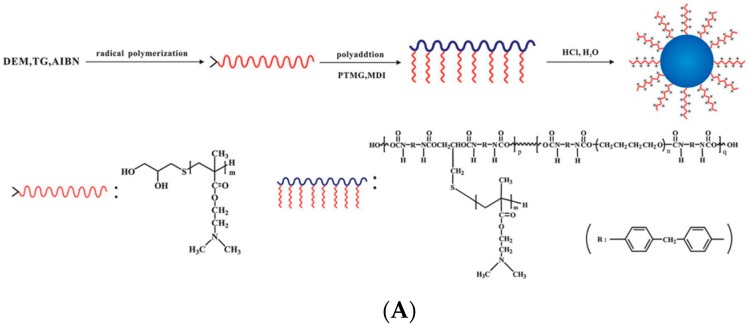

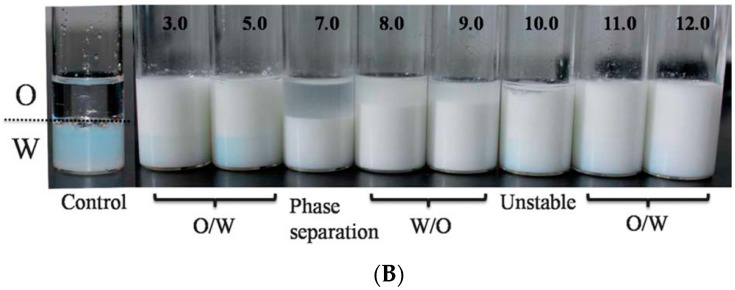
(**A**) Schematic illustration of the synthesis of PU-g-PDEM graft copolymers and their self-assembly in water; (**B**) The effect of solution pH on the appearance of the emulsion (1:1 water/styrene) after standing for 24 h at 25 °C. Adapted with permission from [104]. Copyright 2013, Royal Society of Chemistry.

**Figure 12:**
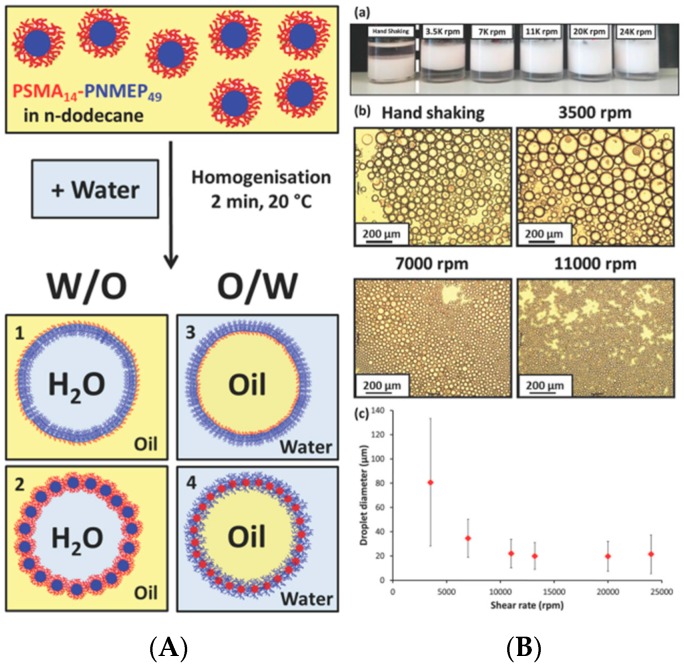
(**A**) Schematic representation of the four possible types of emulsions which could form as a result of homogenizing the poly(stearylmethacrylate)–poly(N-2-(methacryloyloxy)ethylpyrrolidone) (PSMA_14_–PNMEP_49_) nanoparticles prepared in n-dodecane with water; (**B**) (**a**) Digital photographs of Pickering emulsions prepared using PSMA_14_–PNMEP_49_ nanoparticles at various shear rates. Oil-in-water emulsions are formed in all cases, except when hand-shaking is used; this latter approach results in a water-in-oil emulsion instead; (**b**) Optical microscopy images recorded for the droplets prepared via hand-shaking, or via homogenization at 3500 rpm, 7000 rpm or 11,000 rpm (scale bar = 200 µm); (**c**) shear rate dependence for the mean droplet diameter (as determined by laser diffraction) for emulsions prepared using PSMA_14_–PNMEP_49_ spherical nanoparticles as the sole emulsifier. The error bars represent the standard deviation of each mean volume-average droplet diameter, rather than the experimental error. Adapted from [105]. Published by The Royal Society of Chemistry.

In addition to the rigid particles as stated above, soft particles have also been applied to prepare Pickering emulsions. Among them, self-assembled objects have shown great promise to stabilize the oil-water interface [97,98,99,100] and control the stability of Pickering emulsions [101,102]. Fujii et al. [103] synthesized poly[(ethylene oxide)-block-glycerol monomethacrylate-block-2-(diethylamino)ethyl methacrylate] (PEO-PGMA-PDEA) triblock copolymers, which could be dissolved in aqueous solution to form micelles. After further cross-linking the PGMA blocks using succinic anhydride (SA), the spherical shell cross-linked (SCL) micelles were used as emulsifiers. At high pH value, the micelles possessed particular amphiphilicity as a balance of hydrophobic PDEA core and hydrophilic PEO corona. They were able to stabilize W/O emulsions. At low pH, the hydrophobic PDEA core also became hydrophilic, which significantly influenced the amphiphilicity of micelles. Consequently, micelles detached from the oil-water interface and phase separation of the emulsion happened spontaneously (Figure 10). Ma and coworkers [104] used polyurethane (PU)-based nanoparticles as emulsifiers to control the Pickering emulsions. The nanoparticles were formed by self-assembly of an amphiphilic PU-based grafted copolymer which was synthesized by grafting poly(2-(dimethylamino)ethyl methacrylate) (PDEM) side chains on PU main chain. PDEM is a pH-responsive polymer that is hydrophilic at low pH value and hydrophobic at high pH. As shown in Figure 11, a stable O/W emulsion was formed at a pH range from 3 to 5. Upon changing the pH by the addition of base into the emulsion, PDEM was deprotonated and the particles tended to be wetted by the oil phase. The emulsion was inverted into a W/O type at pH 8–9. With further increase of pH value to 11–12, the particles exhibited hydrophilicity again because of the adsorption of the hydroxyl ions on the particle surfaces. Phase inversion occurred once again and O/W emulsions were formed. Cunningham et al. [105] prepared a kind of nano-object consisting of poly(stearylmethacrylate)–poly(N-2-(methacryloyloxy)ethylpyrrolidone) (PSMA–PNMEP) block copolymer by RAFT dispersion polymerization. Spherical nanoparticles were exclusively obtained with increasing degree of polymerization of PNMEP and keeping the PSMA block length unchanged. Dynamic light scattering (DLS) study indicated nanoparticles from one of these block copolymers PSMA_14_–PNMEP_49_ with an intensity-average diameter of 25 nm. As shown in Figure 12, a hand-shaking W/O emulsion was formed by using these nanoparticles as emulsifiers. However, the emulsion was finally inverted to O/W type after high-speed homogenization. In principle, when the oil and water phases were homogenized under high shear, the nanoparticles were assumed to break up into individual polymer chains which practically stabilized the oil-water interface as polymeric emulsifiers. Combining examinations of DLS, laser diffraction, and transmission electron microscopy, the authors claimed that the PSMA_14_–PNMEP_49_ nanoparticles underwent an inversion from initial hydrophobic spheres to hydrophilic spheres during high-speed homogenization. 

### 3.2. Microgels

**Figure 13:**
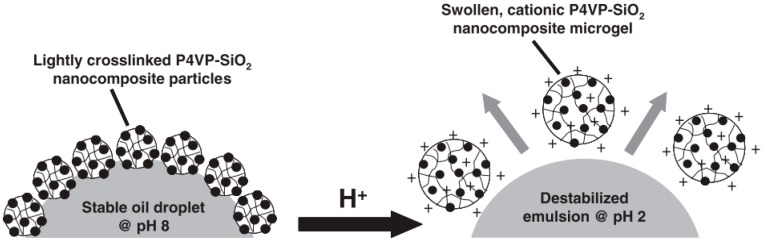
Schematic representation of pH-induced demulsification of an oil-in-water emulsion using lightly cross-linked poly(4-vinylpyridine)/silica (P4VP/SiO_2_) nanocomposites as particulate emulsifiers. Reprinted with permission from [113]. Copyright 2005, WILEY-VCH Verlag GmbH & Co. KGaA, Weinheim, Germany.

**Figure 14:**
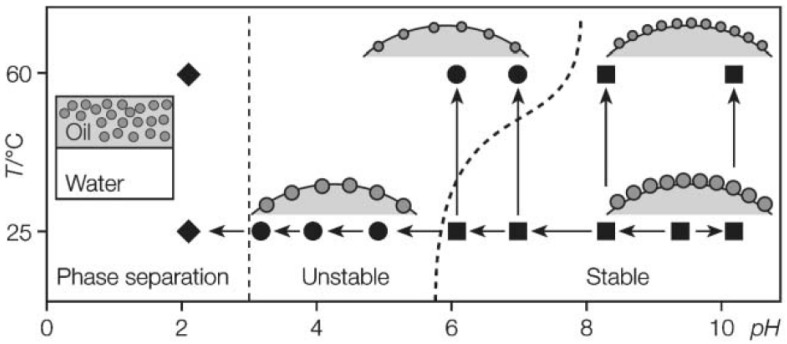
The stabilizing efficiency of poly(N-isopropylacrylamide) (PNIPAM) microgel particles for octanol-in-water emulsions as a function of pH and temperature. ■: Stable, ●: Unstable, ◆: Phase separation. (Arrows indicate the probed transitions). Reprinted with permission from [115]. Copyright 2004, Royal Society of Chemistry.

Microgels are another kind of soft particles used as emulsifiers to stabilize Pickering emulsions. Many examples involving microgels in emulsion systems have been reported in the past few years [106,107,108,109,110,111,112]. Fujii and coworkers [113] synthesized poly(4-vinylpyridine)/silica (P4VP/SiO_2_) microgels for Pickering emulsion. The P4VP/SiO_2_ microgel particles were prepared by polymerizing 4-vinylpyridine monomers in the presence of ultrafine aqueous silica sols. The surface of microgel particles consisted of both hydrophilic silica and hydrophobic P4VP chains, which endowed the particle with certain amphiphilicity. When the microgels were involved in the emulsion system, a highly stable emulsion was obtained at high pH value. The particle amphiphilicity was changed if 4-vinylpyridine residues were protonated at low pH. With stepwise decrease of pH value, the degree of protonation was gradually enhanced and the hydrophobic P4VP became hydrophilic. The microgels no longer stabilized the oil-water interface and desorbed from the interface of emulsion, leading to the destabilization and demulsification of the emulsion (Figure 13). After that, this system was further studied by Binks and coworkers [114]. More details about pH-dependent emulsions with the use of P4VP/SiO_2_ microgels were included. Moreover, the salt effect on the stabilization of Pickering emulsion was also considered in their work. They conclusively demonstrated that the pH value and degree of ionization had significant influences on the stabilization of Pickering emulsions with the use of P4VP/SiO_2_ microgels as emulsifiers. Ngai et al. [115] investigated another smart emulsion system that is responsive to pH and temperature based on PNIPAM microgels. The PNIPAM microgels were prepared by surfactant-free precipitation copolymerization of N-isopropylacrylamide (NIPAM) and methacrylic acid (MAA). Due to the existence of carboxyl groups, the microgel particles were responsive to pH in addition to temperature due to PNIPAM. Hence, the amphiphilicity of microgel particles could be readily tailored by pH or temperature. As shown in Figure 14, when the microgel particles were used as emulsifiers to prepare the Pickering emulsion, stable emulsions were formed at neutral condition at room temperature. With the decrease of pH or increase of temperature, the hydrophobic component of microgel particles was enhanced, which caused destabilization and phase separation of the emulsions. A similar stimuli-responsive behavior was also observed by Brugger and coworkers using PNIPAM–PMAA microgel particles as emulsifiers and interfacial stabilizers [116,117]. 

In addition to the self-assembled objects and microgels, proteins particles have also been exploited to stabilize the Pickering emulsions [118,119,120,121,122,123,124,125,126]. The emulsion size and stability can be well controlled by changing the pH value or salinity of the systems [127,128,129,130].

## 4. Janus Particles

Janus particles are biphasic colloids consisting of two parts with different properties [25]. Different compositions and shapes of the two parts can endow Janus particles with anisotropy in wetting, optical, electrical, magnetic, and catalytic properties. Compared with homogeneous particles, the anisotropic surface wettability of Janus particles can remarkably lower the interfacial tension and afford stronger interfacial adsorption [131]. With this consideration, a few Pickering emulsion systems were investigated by using various amphiphilic Janus particles as solid emulsifiers in recent years [132,133,134]. Analogous to the amphiphilicity of molecular amphiphiles such as surfactants and amphiphilic polymers, the amphiphilicity of Janus particles is also important to determine the stability and type of emulsions in these systems. Surface chemistry and shape of the two parts of Janus particles are two important parameters which have been identified as affecting particle amphiphilicity [46,135]. In this section, we highlight some recent examples of controlling Pickering emulsions by tuning the amphiphilicity of Janus particles from these two aspects: changing surface chemistry and particle shape. 

### 4.1. Surface Chemistry

**Figure 15:**
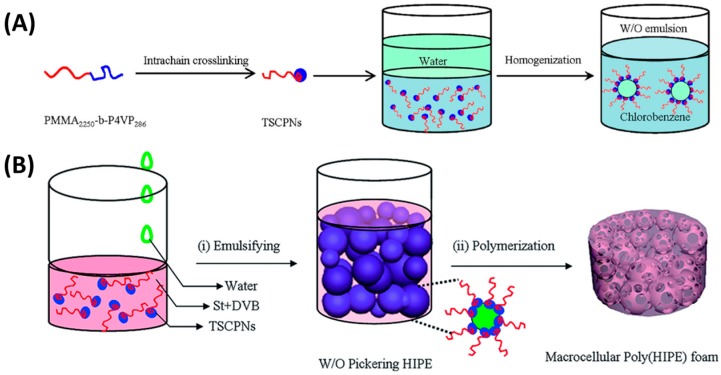
(**A**) Water in Chlorobenzene W/O Emulsions Stabilized by tadpole-like single chain polymer nanoparticles (TSCPNs). Reprinted with permission from [136]. Copyright 2014, American Chemical Society; (**B**) Schematic representation of macrocellular polyHIPE foam templated from TSCPNs stabilized W/O HIPE template. Reprinted with permission from [137]. Copyright 2015, Royal Society of Chemistry.

Great efforts have been devoted to tailoring the amphiphilicity of Janus particles by regulating the surface chemistry. The change in amphiphilicity can facilitate the formation of stable emulsions. Xu and coworkers [136] synthesized tadpole-like single chain polymer nanoparticles (TSCPNs) by intramolecularly cross-linking P4VP block of diblock copolymer PMMA_2250_-b-P4VP_286_. As shown in Figure 15A, this Janus particle contains a cross-linked hydrophilic head and a linear hydrophobic tail, which was able to stabilize the W/O emulsion even at a low concentration of 0.0075 wt%. The obtained Pickering emulsion was used as a medium for heterogeneous reaction. In addition, as shown in Figure 15B, this novel Janus particle was also able to stabilize W/O HIPEs as solid emulsifiers at the appropriate conditions [137]. By solidifying the external oil phase and removing the internal water phase of W/O HIPEs, macrocellular polyHIPE materials with interconnected open-cell structures were obtained which were further extended as supporting matrix for loading Pd catalyst. Chen et al. [138] fabricated Janus nanosheets by crushing the polymer-inorganic hybrid hollow spheres which were synthesized by a self-organized sol-gel process forming silica hollow spheres and subsequent polymer grafting onto the interior side. The Janus nanosheets possess a hydrophilic silica layer and a lipophilic polymer layer, ensuring amphiphilicity as well as interfacial activity for emulsion application. The amphiphilic nanosheets were tolerant against solvents and were successfully used as emulsifiers to stabilize W/O emulsion droplets. Fujii et al. [139] partially modified a gold layer on the silica particles by vacuum deposition to prepare Au-SiO_2_ Janus particles. Due to the hydrophobic property of the Au layer, the surface chemistry of SiO_2_ particles was partly changed. These Janus particles with amphiphilic performance could stabilize O/W Pickering emulsions for a long time. 

**Figure 16:**
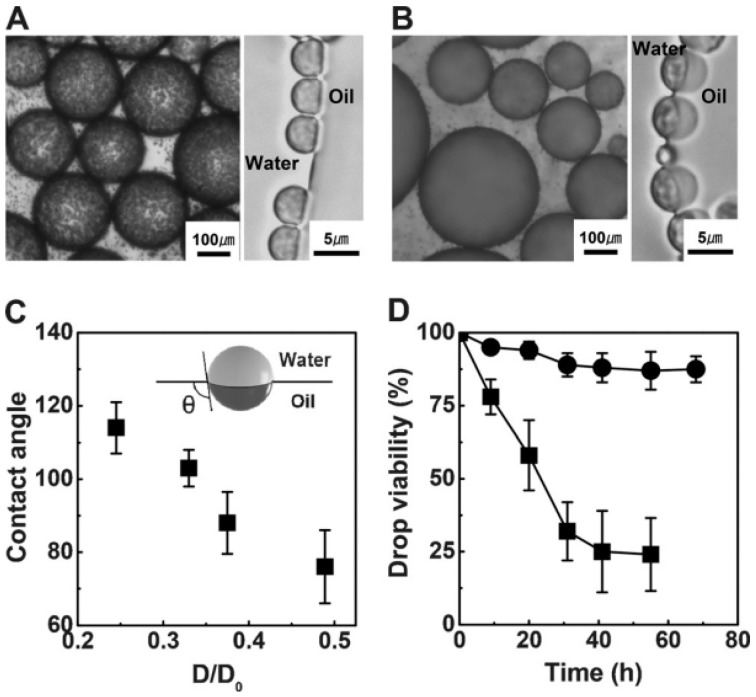
Bright-field microscopy images of the Pickering emulsions stabilized by silica-NP-patched Janus particles with the different degrees of Janusity: (**A**) D/D_0_ = 0.25; and (**B**) D/D_0_ = 0.5. The inset images show adhesion of amphiphilic Janus particles at the hexadecane–water interface; (**C**) Contact angles of silica NP-patched Janus particles at the hexadecane–water interface; (**D**) Viability of Pickering emulsion drops at 50 °C: D/D_0_ = 0.25 (■) and D/D_0_ = 0.5 (●). Reprinted with permission from [135]. Copyright 2016, WILEY-VCH Verlag GmbH & Co. KGaA, Weinheim, Germany.

**Figure 17:**
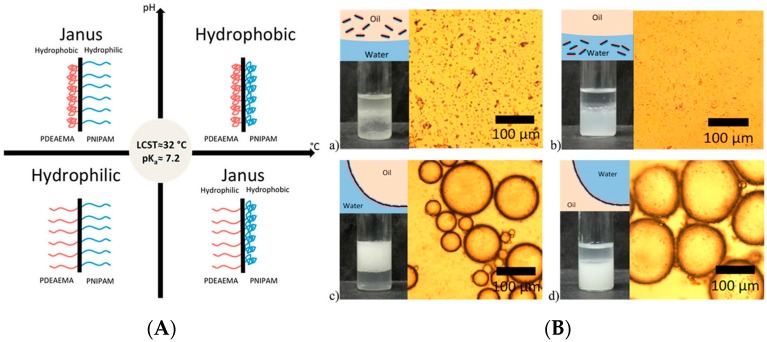
(**A**) Schematic illustration of pH and temperature dually responsive Janus composite nanosheets; (**B**) Emulsification of the immiscible mixture of toluene/water with the PNIPAM/silica/PDEAEMA Janus composite nanosheets. (**a**) Optical microscopy image of the mixture (inset) at pH = 10 and T = 50 °C, no emulsification occurs, the hydrophobic nanosheets dispersible in toluene; (**b**) at pH = 2 and T = 25 °C, no emulsification occurs, the hydrophilic nanosheets dispersible in water; (**c**) a toluene-in-water emulsion forms at pH = 2 and T = 50 °C; (**d**) a water-in-toluene emulsion forms at pH = 10 and T = 25 °C. Adapted with permission from [140]. Copyright 2015, American Chemical Society.

The change of surface chemistry can also facilitate the inversion of different emulsion types. Kim and coworkers [135] synthesized a kind of monodisperse bi-compartmentalized Janus microparticle with high throughput by seed monomer swelling and consecutive polymerization. The hydrophilic silica nanoparticles were patched on one of the compartmented bulbs to endow the Janus particles with amphiphilicity. The amphiphilicity of Janus particles was precisely tailored by controlling the relative dimension ratio of the hydrophobic bulb against the whole particle, which was called “degree of Janusity” as the authors stated. When the degree of Janusity was near 0.25, W/O emulsions were formed. As shown in Figure 16D, with the increase of the degree of Janusity, the change in amphiphilicity enabled the phase inversion from W/O emulsion to O/W emulsion and the stability of the generated emulsion was also enhanced. Zhao et al. [140] grafted the temperature responsive PNIPAM and pH responsive PDEAEMA on two sides of a silica nanosheet to prepare a dually responsive Janus particle. The amphiphilicity of Janus particles could be tailored by changing the pH or environmental temperature. Different types of emulsions were formed and transformed each other by using these Janus particles with tunable amphiphilicity. At high pH and high temperature, Janus particles were completely hydrophobic, which were only dispersed in the toluene phase. At low pH and low temperature, the Janus particles on the contrary were hydrophilic and dispersed in the water phase. With proper change of pH and temperature, the Janus particles showed amphiphilicity and could stabilize the oil-water interface. At low pH and high temperature, O/W emulsions were formed. At high pH and low temperature, W/O emulsions were preferred. The control of phase inversion of emulsions was achieved by a combination of pH and temperature (Figure 17). 

### 4.2. Shape

**Figure 18:**
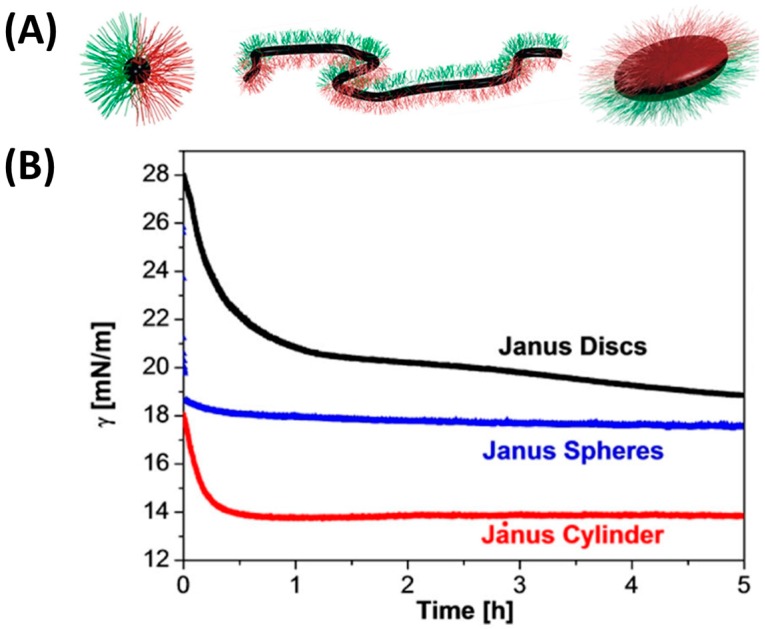
(**A**) Overview of possible Janus particle architectures: Spheres, Cylinders, and Discs; (**B**) Influence of the Janus particle shape on the interfacial tension. Interfacial tension isotherms of solutions of Janus particles in toluene at a water/toluene interface. Adapted with permission from [141]. Copyright 2013, American Chemical Society.

**Figure 19:**
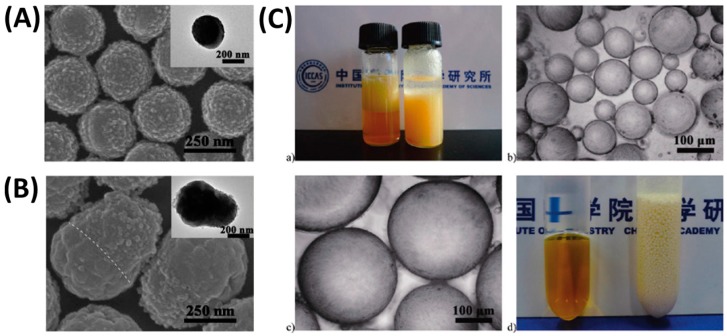
Morphological evolution of the anisotropic composite particles at varied monomer/particle weight ratio: (**A**) 2:1; (**B**) 16:1; (**C**) Janus characteristics of the anisotropic particles. (**a**) Left: a decane-in-water emulsion stabilized with the Janus particle as shown in Figure (**A**); right: a water-in-decane emulsion stabilized with the Janus particle as shown in Figure (**B**); water/decane volume ratio is 2:1, and the particle/water weight ratio is 1:100; (**b**) Optical microscopy images of the decane-in-water emulsion; and (**c**) water-in-decane emulsion; (**d**) Left: immiscible toluene/water mixture; right: a water-in-toluene emulsion stabilized with the Janus particle as shown in Figure 5d. Water/toluene volume ratio is 2:1, and the particle/water weight ratio is 2:1000. Adapted with permission from [46]. Copyright 2012, American Chemical Society.

The shape of the Janus particles is another factor that has a significant influence on their interfacial behavior. Ruhland and coworkers [141] investigated in detail the interfacial behavior of Janus particles with different shapes including Janus spheres, Janus cylinders, and Janus discs by a combination of dynamic interfacial tension measurements and computer simulations. The different adsorption kinetics and equilibrium values of the interfacial tension with the use of different Janus particles were compared (Figure 18). The particle shape is considered to affect the surface activity of Janus particles. The stability and type of Pickering emulsion stabilized by these Janus particles show close dependence on the shape of the Janus particles. Multiple shaped Janus particles have been practically exploited as solid emulsifiers for the formation of stable emulsions and controllable inversion of different emulsion types. A snowman-like Janus particle consisting of a hydrophilic bulb and a hydrophobic bulb was synthesized based on the seeded polymerization technique by Kim and coworkers [142]. Stable emulsions of spheres, ellipsoids, and cylinders could be formed due to the substantial absorption of these amphiphilic Janus particles at the O/W interface. The inversion of different emulsion types was also achieved by changing the relative ratio of hydrophilic and hydrophobic bulbs in the snowman-like particles by Liu and coworkers [46]. Different from the seed polymerization technique, the snowman-like Janus particles in their study were prepared by extruding a lobe based on swelling the polymeric core from a spherical core-shell structure. By tuning the monomer/particle weight ratio, the relative size ratio of the hydrophilic inorganic part and the hydrophobic polymeric lobe could be tailored. When the Janus particles with a large hydrophobic polymeric lobe were used to stabilize the Pickering emulsion, a W/O emulsion was formed. With the decrease of hydrophobic lobe size, the emulsion was inverted into an O/W type (Figure 19). 

**Figure 20:**
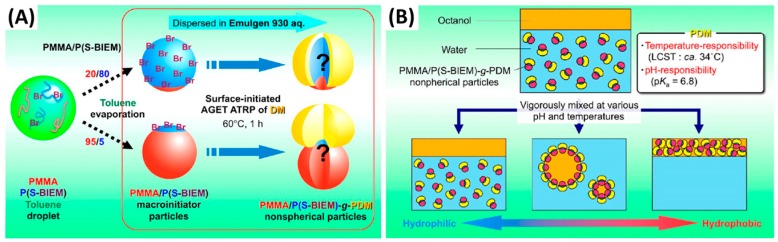
(**A**) Preparation of mushroom-like Janus polymer particles by site-selective surface-initiated activator generated by electron transfer (AGET) ATRP in aqueous dispersed systems; (**B**) The scheme of whole behaviors of Pickering emulsion formed by stimuli-responsive “mushroom-like” Janus polymer particles. Adapted with permission from [143]. Copyright 2014, American Chemical Society.

**Figure 21:**
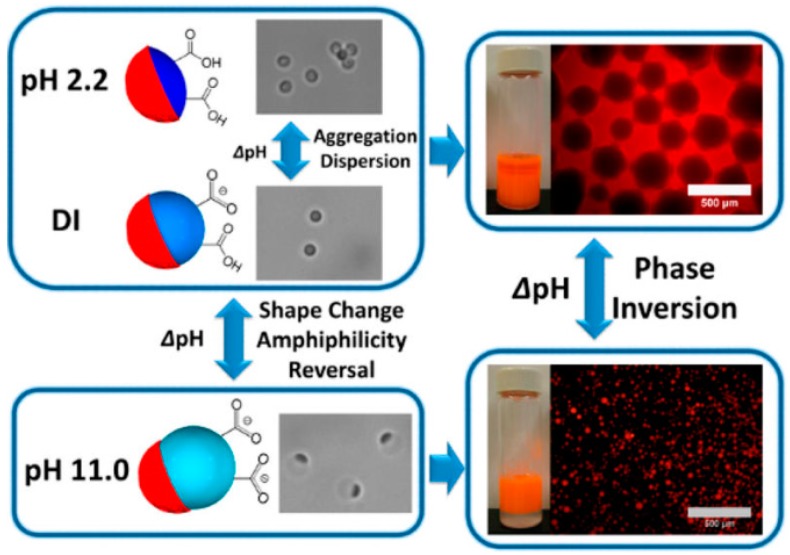
Janus particles change their aggregation/dispersion behavior and also transform into different shapes in response to pH changes. Janus particles with tunable amphiphilicity can stabilize different types of emulsions (oil-in-water and water-in-oil). Reprinted with permission from [144]. Copyright 2014, American Chemical Society.

Another novel “mushroom-like” Janus particle [143] was reported to control the stability of Pickering emulsions by Yamagami and coworkers (Figure 20). The poly(methyl methacrylate)/poly(styrene-2-(2-bromoisobutyryloxy)ethyl methacrylate)-graft-poly(2-(dimethyl amino)ethyl methacrylate) PMMA/P(S-BIEM)-g-PDM Janus particles were synthesized by using a surface-initiated activator generated by electron transfer (AGET) ATRP to graft PDM on the P(S-BIEM) side of the composite particles based on phase separation under solvent evaporation. The shape anisotropy of the Janus particles could be precisely tailored by changing the size of the P(S-BIEM) side, which was conducted by controlling the P(S-BIEM) content in the composite particles. The introduction of PDM enables the Janus particles to be dually responsive to pH and temperature. Janus particles with controllable shapes were used as solid emulsifiers to stabilize 1-octanol-in-water emulsion. The stability of the emulsion could be controlled by altering the temperature and pH. Tu et al. [144] fabricated a shape switchable Janus particle to control the phase inversion of Pickering emulsions. The Janus particles were synthesized by seeded emulsion polymerization and subsequent acid hydrolysis. Due to a pH-responsive ability, the particle part with acrylic acid blocks were swollen at high pH and deswollen at low pH, thus making the particle shape controllable by pH. As shown in Figure 21, the Janus particles remained oblate-like or almost spherical shape at pH 2.2 or in a deionized water medium, which favored the formation of W/O emulsions. With increasing pH to 11.0, the shape of Janus particles was transformed into a dumbbell. The interfacial activity of the Janus particles also changed with the shape change, inducing the emulsions with different types. W/O and O/W emulsions could be reversibly switched by adding a small amount of acid or base in the emulsion system. This study was claimed as the first example of transitional phase inversion of Pickering emulsions by dynamically tuning the shape to reversibly change the amphiphilicity of the Janus particles.

## 5. Conclusions

In this article, we comprehensively reviewed recent progress in tuning the amphiphilicity of particles for controllable Pickering emulsions. The whole map of amphiphilicity regulation was presented on the basis of particle species. Rigid particles can be used to control the stability and type of Pickering emulsion by molecular adsorption or chemical grafting of functional substances. Soft particles including self-assembled objects and microgels were also highlighted for the control of stabilization and destabilization of emulsions. Anisotropic Janus particles most likely bridging two immiscible phases together were highly emphasized. Their amphiphilicity, referring to the interfacial activity, was tuned by changing the particle surface chemistry or shape. From the aspect of amphiphilicity, this review gives new bearing on the preparation of controllable emulsions, though most examples did not correlate the controllable Pickering emulsions with the amphiphilicity of the particles. It is believed that this concept will open a new avenue to guide the preparation of functional materials based on emulsion technique. However, there are still existing opportunities and challenges in this area. Novel particles are expected to be synthesized for the preparation of complex Pickering emulsions including double emulsions and HIPEs, which requires the particles to stabilize at least two oil-water interfaces at the same time. More advanced emulsions such as W/W or O/O emulsions are hoped to be exploited by nanoparticles with ideal amphiphilicity. In addition, Pickering emulsion systems as a result of tuning amphiphilicity may be manipulated by biological actuators, which will meliorate the toxicity issue in enzymatic and clinical applications.
